# Supplementary material for: Patterns of Intron Gain and Loss in Fungi
Source: PLoS Biol. 2004 Nov 30;2(12):e422. doi: 10.1371/journal.pbio.0020422 (PMC532390; doi:10.1371/journal.pbio.0020422)
Supplement: Table S1 — Also available at http://genes.mit.edu/NielsenEtAl/. (4.3 MB ZIP). [file pbio.0020422.st001.zip › NielsenEtAl/html/956.html]

AN1632.1.NCU00188.1.MG06393.1.FG05547.1


```
 CLUSTAL W (1.82) Multiple Sequence Alignments - Introns Inserted


Sequence 1: NCU00188.1	932 aa
Sequence 2: MG06393.1	982 aa
Sequence 3: FG05547.1	944 aa
Sequence 4: AN1632.1	935 aa
Alignment Length: 1005 aa
Number Identitical Residues: 349 aa
Alignment Score (without introns) 17116


MG06393.1 	MADR-----SARRARPGDDSVGQFVIGAEIGKGSFAQVYMGKHK0VSGAAVAIKSVELAR
NCU00188.1	MSDRTSASSSSRRTKQNYDAIGSFVIDQEIGKGSFAKVYLGRHK0VTGALVAVKSVELAR
FG05547.1 	MAGPQESSTSSGSRKSGSRAVGQFNIGSEIGKGSFAQVYLGWHK0ETKAAVAIKSVELER
AN1632.1  	MASTPSKSSRSSKEAYSEVAIGPYTRLDHIGKGSFATVYRGVQT0KSRTFVAIKSVNLGK
          	*:.  . :: :     .  ::* :    .******* ** * :.  : : **:***:* :

MG06393.1 	LNKKLKENLYGEINILKTLRHPHIVALHDCVESATHINLMMEYCELGDLSLFIKKREKLS
NCU00188.1	LNKKLKENLYGEIQILKTLRHPHIVALHDCVESSTHINLIMEYCELGDLSLFIKKRDKLI
FG05547.1 	LNKKLRENLYSEIQILKTLRHPHIVALHDCIESTSHINLIMEYCELGDLSLFIKKREKLA
AN1632.1  	LNRKLKDNLAMEIDILKYLLHPHIVALLDCLETNSHIHLIMEYCALGDLSQFIKRRDSLK
          	**:**::**  **:*** * ******* **:*: :**:*:**** ***** ***:*:.* 

MG06393.1 	TNPATHDMARKYPNVPNSGLNEVVIRHFLKQLSSALKFLRESNLVHRDVKPQNLLLLPSP
NCU00188.1	TNPYTHDLARKYPVYPNAGLNEVVTRHFLKQLASALQFLRAGDFVHRDVKPQNLLLLPSP
FG05547.1 	THPATHDMARKYPSMPNSGLHEVVIRHFLKQLTSALEFLRSKNYVHRDVKPQNLLLLPSQ
AN1632.1  	DHSYTRHMISKYPNVSGGALNEVIVRHFLKQLASALRFLRDKNLIHRDIKPQNLLLCPAP
          	 :. *:.:  ***  ....*:**: *******:***.***  : :***:******* *: 

MG06393.1 	EFREVNKLARPILT---ASQDSLVPVAGLASLPMLKLADFGFARVLPSTSLAETLCGSPL
NCU00188.1	HMMANNKTAKHIMS---GSYDSFTPAAGLASAPMLKLADFGFARVLPSTSLAETLCGSPL
FG05547.1 	PFRDQ--RSRPVMQ---ASQDSLIPISGLASLPMLKLADFGFARVLPSTSLADTLCGSPL
AN1632.1  	KPAPTQSEAEPQIVPLKGSETSFTPAVGLETLPLLKLADFGFARSLPATSLAETLCGSPL
          	     .. :.  : . ..*  *: *  ** : *:********** **:****:*******

MG06393.1 	YMAPEILRYERYDAKADLWSVGTVLFEMIVGRPPFRASNHVELLRKIEAAEDVIKFPRET
NCU00188.1	YMAPEILRYEKYDAKADLWSVGTVLYEMVTGRPPFKASNHVELLRKIESSGDVIKFTRES
FG05547.1 	YMAPEILRYERYDAKADLWSVGTVLYEMSTGRPPFRARNHVELLRKIEAAEDVIKFPREV
AN1632.1  	YMAPEILRYEKYDAKADLWSVGTVLYEMVVGKPPFRATNHVELLRKIETARDRIKFPEEN
          	**********:**************:** .*:***:* **********:: * ***..* 

MG06393.1 	TISSEMKGLTRALLKRNPVERISFENFFAHPVIISSIPGLVEDDIPKPEASEQRSSSKDT
NCU00188.1	VVSQEMKGLIRALLKKNPVERISFEDLFNHPVVTEPIPGLVEDDIPKP---PRRRSLKEE
FG05547.1 	SITPELKALIRSLLKRSPVERLSFENFFTHQVVTSEIPGLVEDDIPKS----LRQESRDP
AN1632.1  	PASDDIKALVRGLLKFNPVVRMTFADFFENDIITGPIPGLAAEDVPIP----DRPPSPEV
          	  : ::*.* *.*** .** *::* ::* : ::   ****. :*:* .     *    : 

MG06393.1 	RAASKSDDPIASPRKYSFRRHPTDNDQIRDQFRRVEPPSSAAESAPSRQTSAFSGIAAEA
NCU00188.1	RPVSRAEDSLVPSRKQSLRKDLADRE---GAPQTAGPSSPKPRRSSPLATPNEPVEISKP
FG05547.1 	RSAFQSGSPSLSSRSPRQTGHQSPTE----ALVSRSPRDQQPR-SPQVGSP------GGS
AN1632.1  	IPTESAEPQSVSDTIYPRNNDAEPKE------DRYPPPHRYVPHNDRPQTP------TSS
          	 ..  :     .        .    :          *            :.        .

MG06393.1 	RKQAAAEASARTGQSSRNEPGDNLVPRRPQAQPSTSAPSKPGLYEERRRGISNASLNRSN
NCU00188.1	NYFQIPPREDRLSYSPRKEAADGLGIKRPQVQPSTSAPTRPSSYVDRRHRSSNASLRAPV
FG05547.1 	RYARRSNESQRTTGNSPREGGEGLGIRRPVAQHAMTAPVQQVAYD---------SVTGRN
AN1632.1  	SPMRRTRSGDRP--SSAKQIPPAAAPPRPEAVSHATAPGRQ-------------ELIGRH
          	     .    *   .. .:        ** .    :** :              .:    

MG06393.1 	RESSSPTSAALANDSARAPPQQTSRRVQAEEREKAAQDVAFERDYVVVEKKHVEVNAFAD
NCU00188.1	REANPPP-----NDVTRTKPRGMGTKPMTEE-ERAAQDIAFERDYVLVDKKHLEVNALAD
FG05547.1 	RASPPTS----LLDQVRRN-RALSNPPITEE-ERAAQDVALEREYVVVERRHVEVNALAD
AN1632.1  	SSEQQAP---------GTVPVYKNVDKLKEERELAAQEVAFERDYVVVEKRAVEVNAFAD
          	     ..            .   .     ** * ***::*:**:**:*::: :****:**

MG06393.1 	EMAANPRLGGQGTPLSPKSGQIVRRATQQGNPTSTTGAIPAAPSRTMQIAQGT------Q
NCU00188.1	QISMYPQQ-----PQSPKSAQIVRRATQQGSPTSTSGAVPSPPSRALQLAQGH------S
FG05547.1 	ELAANEKLG----DASQRSGPITRRYTQQGAPTSTTGAISTPYSRNALATQPR------H
AN1632.1  	QLAYDPRFH------GKQTGALPRRQTTPALPTTQASSQNSP-RKAVQVVSGRSRADSAH
          	:::   :        . ::. : ** *  . **: :.:  :.  :    ..  : :.:: 

MG06393.1 	RHYHERGTSLSASPGSTASFITKAIQDASLRLLGIKYPAG---LTKGASPPELYNPYPAY
NCU00188.1	RQ-GSYDKALGTSPSKATSVISKAIQDASLRLFGFKYAPHL--LSKGPSPVPMYNPFPTY
FG05547.1 	DRKSSYEKSLSASPGSASSAISKAIQDASLRLFGFKVPPLR--ASPKGPSPPLYQAFPTY
AN1632.1  	NRHASYERRYGQSPTSATSAISKALNMASGRLFGVGFSPPLNIAKGGRSPPLAYNPFPAY
          	 :  .     . ** .::* *:**:: ** **:*.  ..  .  .   ..   *:.:*:*

MG06393.1 	PTPSPPVGLISSGKQSTP-VDEDARVAQLIEEHATRSDVVYGFAEVKYKQLVPLAPSAEH
NCU00188.1	PAPNTPTGLISDGKQGTP-VDEDSRVAQCIEDHATRSDVVYGFAEVKYKQLVPLAPSMDH
FG05547.1 	PTPQAPVGLLGDGRNVQG-TDEDGKAAQTIEELATRSDCVYGFAEVKYKQLVPLAPSADH
AN1632.1  	PTGHAGLLTMGDGARAQASPDEDTRTVKELEECATRSDVVYGFAEVKYKQLIPLAPSSQS
          	*:  .    :..* .   : *** :..: :*: ***** ************:***** : 

MG06393.1 	GLGGTTLEDMPTGEEDVLTVEAIVSLSEEALVLYVKALTLLAKSMDIASLWWARKNRGDQ
NCU00188.1	GLGGAPIEKS--AEEDGLTAEAIVSLSEEALVLYVKALTLLAKSMDIASLWWSRKNKVES
FG05547.1 	ILGGLEPEQL-VNEEDGLTVEAIVALSEEALVLYVKSLTLLARAMDIASLWWSKKSRGDT
AN1632.1  	YNSPNAPGTN--FEEGELTPDAAVTLSEEALVLYVKALSLLAKSMDIAGAWWMRKNRAEA
          	  .          **. ** :* *:***********:*:***::****. ** :*.: : 

MG06393.1 	ANNALSSTRDSVNTQTLSLRINSAVQWVRSRFNEVLEKAEVVRLRLMDAQKRLPDDHPSH
NCU00188.1	TNSITSAARDSANSEALALRINGAVQWIRSRFNEVLEKAEIVRLKLAEAQKRLPEDHPSH
FG05547.1 	GTGLSAAA-----AQTVVQRINAVVQWVRQRFNEVLEKSEIVRLKLTEAQKQLPDDHPSH
AN1632.1  	LGESSFSR-----LEPVSIRINKVVQWVRSRFNEVLEKAEFVRLKLLEAQRRLPTDHPSH
          	      :       :.:  *** .***:*.********:*.***:* :**::** *****

MG06393.1 	PSNHPQGSESVNGAS-AEGVFLTVGVTAEKLMYDRALEMSRTAAINEITNEDLAGCEISY
NCU00188.1	PNNHANDSTALNSLS-TVGVFLSEGISAERLMYDRAIEMSRAAAINEIANEDLPGCEISY
FG05547.1 	PSNHGTESIASSAGSPTKQVYLTPGISAEKLMYDRALEMSRAAAIDEVTNENLSGCEISY
AN1632.1  	PNNLSLES-AGSGTS--VDVMVSPGVTAEKLMYDRALEMSRAAAINELTSEDLPGCEIAY
          	*.*    * : .. *    * :: *::**:******:****:***:*::.*:*.****:*

MG06393.1 	VTAIRMLEAVLDNDED--APKRRLSANMDDSGGDGEDGH-AE~INADDQQAVQKM1IHMI
NCU00188.1	ITAIRMLEAVLDQDDDH-LPKRKVSGASKEEKTGANELS-DE~MNNEDKQVVQ--~----
FG05547.1 	ITAIRMLEAVLDNDEGSGSETRRLSTGKEAEREAVKEVSGGE~LDSDEEAHVRK-~----
AN1632.1  	VTAIRMLEAVLETDEVQPSGKGTDKVTLDGVQGEEREVLLKL1VSKIRMRLATLR~----
          	:**********: *:     .   .   .      .:      :.      .        

MG06393.1 	RS~RLTSVRSKVRMISNASKAQQQSQPQSLIRRRSGDVTPRSVPSYSS-
NCU00188.1	--~--------------------NGEPTSVLVQNKASLY----------
FG05547.1 	-R1RLAAVRKKQQMIAEAN-SKTNLVYQQAVRRRSGDMTPRSVPSHASS
AN1632.1  	-Q~KLAVLAKRQTPPSSGKMIPSNLAHATPATPATGMATPR--------
          	     :   .     :...    :          ..   .
```
